# Action Potential: A Vortex Phenomena; Driving Membrane Oscillations

**DOI:** 10.3389/fncom.2020.00021

**Published:** 2020-03-18

**Authors:** Raghottam M. Sattigeri

**Affiliations:** Department of Physics, Faculty of Science, The Maharaja Sayajirao University of Baroda, Vadodara, India

**Keywords:** action potential, nerve membrane, neurophysics, vortex theory, membrane oscillator theory, biophysical model

## Abstract

Hodgkin-Huxley (HH) model has been one of the most successful electrical interpretation of nerve membrane which led to revolutions in the field of computational neuroscience. On the contrary, experimental observations indicate that, an Action Potential (AP) is accompanied with certain physiological changes in the nerve membrane such as, production and absorption of heat; variation of axon diameter, pressure and length. Although, in the early 1900's a Pressure Wave Theory was proposed by E. Wilke, but, due to lack of sophisticated experimental techniques it was left uncharted. Until recently, when Heimburg-Jackson, Hady-Machta and Rvachev, independently proposed Soliton Theory (thermodynamic interpretation of nerve membrane), Mechanical Surface Waves theory (electro-mechanical interpretation) and Rvachev Model (mechano-electrical activation of voltage gated sodium ion channels) respectively; encouraging a deviation from the traditional HH interpretation with justification for the physical changes in the nerve membrane observed experimentally. But, these theories lead to a “*hit and miss*” scenario because, they do explain certain features (increase/decrease in axon diameter) but miss to explain, *correlation* between the strength of stimuli and spike rate of AP. Bio-physical models of nerve membrane are thus important for enhancing our understanding regarding the governing dynamics of neural activities encompassing the experimental observations. A novel theory is proposed here which, unravels *vortex ring* formation due to ion currents in the intracellular and extracellular region leading to variation of pressure causing the increment/decrement in axon diameter. These formations manifest as *membrane oscillations* which are used to establish a *correlation* between the strength of stimuli and spike rate of AP. The theory proposed in this paper, brings a paradigm shift in our understanding of neural dynamics from a thorough bio-physical and physiological perspective with promising applications.

## 1. Introduction

**Figure 1:**
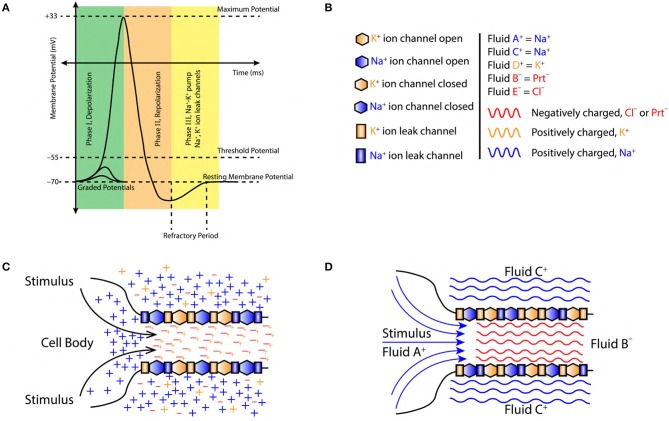
**(A)** A typical AP, divided into three major phase, (I) Depolarization, (II) Repolarization, and (III) Refractory period (active Na^+^−K^+^ pump and Na^+^, K^+^ ion leak channels). **(B)** Symbols used in figures, sodium ions (blue color, Na^+^), potassium ions (orange color, K^+^), chlorine ions (red color, Cl^−^) and negatively charged proteins (red color, Prt^−^). Fluid constituting positive ions are approximated as fluid A^+^, C^+^ and D^+^ with a net positive charge (blue and orange color fluid, respectively). Similarly, fluid constituting negative ions are approximated as fluid B^−^ and E^−^ with a net negative charge (red color fluid). **(C)** Initiation of AP at the axon hillock in the cell body due to accumulation of sodium (Na^+^) ions (blue color +) resulting from a stimuli. The axoplasmic fluid is filled with potassium (K^+^) ions (orange color +) which tend to linger around the negatively charged proteins (Prt^−^) (red color −). **(D)** Charged fluid approximation (based on section 2.2) for the initiation of AP at axon hillock in the cell body.

In an attempt to understand the governing dynamics of neural activity, several models have been designed (Hodgkin and Huxley, 1952b; Harmon and Lewis, 1966; Debanne, 2004; Heimburg and Jackson, 2005, 2007; Bean, 2007; Kim et al., 2007; Andersen et al., 2009; Rvachev, 2010; Fields, 2011; Tyler, 2012; Debanne et al., 2013; Mueller and Tyler, 2014; Shrivastava, 2014; El Hady and Machta, 2015; Engelbrecht et al., 2016). These models are based on different interpretations of Action Potential (AP) (Figure 1A) which govern the manifestation of microscopic functions of a neuron on a macroscopic scale in the brain. Hodgkin and Huxley (HH) (Hodgkin and Katz, 1949; Hodgkin and Huxley, 1952a,b; Hodgkin, 1964a,b) established first model which interprets AP as a purely *electric* phenomena. Although this model led to revolutions in the field of computational neuroscience, it could not successfully incorporate other physiological features observed experimentally which accompanied AP in a neuron (Hill, 1950; Abbott et al., 1958; Julian and Goldman, 1962; Biondi et al., 1972; Hill et al., 1977; Iwasa and Tasaki, 1980; Iwasa et al., 1980; Tasaki and Iwasa, 1982; Terakawa, 1985; Tasaki et al., 1989; Tasaki and Byrne, 1992a,b; Galbraith et al., 1993; Tasaki, 1999). Decades after the seminal HH interpretation, Soliton Theory (ST) by Heimburg-Jackson (Heimburg and Jackson, 2005, 2007), Mechanical Surface Wave (MSW) by Hady-Machta (El Hady and Machta, 2015) and the Mechano-Electrical activation of sodium channels in Rvachev Model (Rvachev, 2010) addressed AP from a physiological perspective which incorporated the empirical observations. Although these theories explain the phenomena of AP to a great accuracy but, not in entirety. New approximations are thus necessary for a thorough interpretation of AP which will facilitate the development of more accurate neuronal models and further our understanding about the nature of nerve membrane which lies at the foundation of various neuronal activities resulting in a complex brain function.

The model proposed by Heimburg-Jackson, suggests that, AP exhibits a *solitonic* behavior i.e., AP exerts a pressure perpendicular to the surface of nerve membrane and travels down the axon without any dissipation (Heimburg and Jackson, 2005, 2007). Also, this model treats generation of heat in the nerve membrane associated with the event of AP in a thermodynamic regime. Overall, this model does explain and indicate toward an increment in the axon diameter which is accompanied by the absorption and emission of heat by the nerve membrane but, it does not explain the decrement in axon diameter which follows subsequently. The thermodynamic and solitonic interpretation is quite useful for the investigation of collisions between two AP in a nerve membrane (Budvytyte et al., 2014).

The electro-mechanical model proposed by Hady-Machta suggests that, AP is driven by electric constriction of a *dielectric* axon which indicates *electro-mechanical* origin of AP (confirmed computationally) (El Hady and Machta, 2015). This model successfully explains the origin of mechanical and thermal variations in the nerve membrane but, does not explicitly relate, the contribution of ion currents to the resulting mechanical changes in the nerve membrane. On the other hand, a rather versatile model proposed by Rvachev (2010), treats AP as a phenomena driven by the *mechano-electrical* activation of voltage gated sodium channels. This model successfully addresses physiological changes in nerve membrane (such as variation in mechanical, optical and thermal properties) to some extent the role of ion currents in the event of an AP but does not correlate, the spike rate (frequency) of AP with the strength of stimuli.

The models proposed so far result from, purely electric/mechanical/thermodynamic/electro-mechanical/mechano-electrical interpretation of the nerve membrane during the event of an AP. This has led to a broad bifurcation of theoretical models into two regimes, (i) electrical and (ii) non-electrical. In this paper, a new interpretation of nerve membrane pertaining to the event of AP is proposed which brings a paradigm shift in our understanding of nerve physiology and the underlying dynamics. Retaining the thermodynamic/electro-mechanical/mechano-electrical treatment of nerve membrane (Heimburg and Jackson, 2005, 2007; Rvachev, 2010; El Hady and Machta, 2015) to address the absorption and emission of heat during the transport of AP, we focus on the role of ion currents across the nerve membrane during the event of an AP. The resulting interpretation unravels a *vortex* phenomena governing the formation and propagation of an AP through the axon due to *peristaltic* motion of the nerve membrane. Mechanical changes in the axon (such as variation of axon pressure, diameter and length) due to stress and strain result from, the pressures built-up in the intracellular and extracellular region due to *vortex ring* formation during depolarization (in the intracellular region) and repolarization (in the extracellular region) states, respectively. The fascinating outcome of this theory is that, the *vortex ring* formation drives nerve membrane into *oscillations* about its equilibrium position. This prediction enables us to establish a *correlation* between the strength of stimuli and spike rate (frequency) of AP, which has not been addressed thoroughly so far. Also, the proposed model is more realistic in terms of its broad umbrella which encompasses across every important facet of the nerve physiology pertaining to AP.

## 2. Vortex Theory (VT)

### 2.1. Hypothesis

*Ion currents across the nerve membrane during, depolarization and repolarization phase of an AP form* ***vortex ring***.

### 2.2. Assumptions

Certain approximations and schematics will be followed to establish the VT for AP. These approximations neglect the complexities of molecular cell biology and are rather addressed from a macroscopic *bio-physical* perspective. This will serve as a *prelude* to the VT.

**Figure 2:**
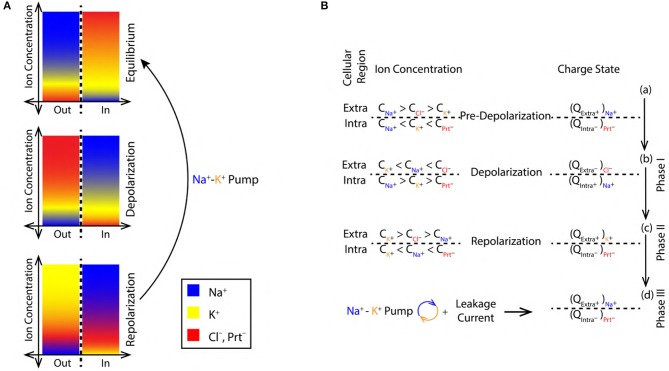
**(A)** Color coded ion concentration gradient across (IN and OUT) the nerve membrane at, equilibrium (maintained by the Na^+^-K^+^ pump and ion leak channels), depolarization and repolarization. **(B)** Ion concentration schematics representing the net charge state in the intracellular and extracellular regions during, pre-depolarization 2.3, depolarization 2.3 and repolarization 2.3 state.

Elementary physics suggests that, a charge in motion gives rise to current (Halliday et al., 1995); ion currents across the membrane during, generation and propagation of AP are one such example in biological systems. These currents have not been addressed explicitly through the, ST, and MSW models discussed previously (section 1). It is necessary to incorporate this (ion currents) key feature of a nerve membrane to completely justify the phenomena of AP from a physiological perspective. For this purpose, certain approximations are made as follows:

1. The intracellular and extracellular regions of a neuron are made up of incompressible fluids (Figure 1D).
2. These fluids are composed of charged ions (sodium Na^+^, potassium K^+^, chlorine Cl^−^) and proteins Prt^−^ (Figures 1B,C). The net charge of the fluid is governed by the concentration of ions (Figure 2B). This implies, positive/negative fluids are dominated by the positive/negative ions, respectively (Figure 1C).
3. The ion currents across the nerve membrane are approximated as, flow of charged fluid through the nerve membrane.
4. The forces governing fluid flow across the nerve membrane are, diffusive and coulombic in nature.
5. Interactions with charged entities (other than those mentioned here) in the extracellular and intracellular region are neglected.
6. The nerve structure is unipolar with the shape of axon post-axon hillock following cylindrical symmetry.

### 2.3. Ion Schematics

#### 2.3.1. Pre-depolarization

The concentration of Na^+^, K^+^, and Cl^−^ ions in the extracellular region before depolarization are, ${\text{C}}_{{\text{Na}}^{+}}$, ${\text{C}}_{{\text{K}}^{+}}$, and ${\text{C}}_{\text{C}{\text{l}}^{-}}$, respectively. These concentrations are related as, ${\text{C}}_{{\text{Na}}^{+}}$ > ${\text{C}}_{{\text{Cl}}^{-}}$ > ${\text{C}}_{{\text{K}}^{+}}$ (Figure 2B). Similarly, the concentration of K^+^, Na^+^ ions and proteins with net negative charge (Prt^−^) in the intracellular region are, ${\text{C}}_{{\text{K}}^{+}}$, ${\text{C}}_{{\text{Na}}^{+}}$, and ${\text{C}}_{{\text{Prt}}^{-}}$, respectively. They are related as, ${\text{C}}_{{\text{Na}}^{+}}$ < ${\text{C}}_{{\text{K}}^{+}}$ < ${\text{C}}_{{\text{Prt}}^{-}}$ (Figure 2B). Thus, in the pre-depolarization state, the extracellular region is electro-positive and the intracellular region is electro-negative governed by the concentration gradient (as shown in Figure 2A). This is represented by the net charge state, in the extracellular region (by the virtue of Na^+^ ions) and in the intracellular region (by the virtue of Prt^−^) as, ${\text{Q}}_{{\text{extra}}^{+}}{|}_{{\text{Na}}^{+}}$ and ${\text{Q}}_{{\text{intra}}^{-}}{|}_{{\text{Prt}}^{-}}$, respectively (Figure 2B).

#### 2.3.2. Phase I, Depolarization

During depolarization (Figure 1A), due to diffusion of Na^+^ ions into the intracellular region (Lodish et al., 1995; Purves et al., 2008; Hall, 2010), the ion concentrations in extracellular and intracellular region change to, ${\text{C}}_{{\text{K}}^{+}}$ < ${\text{C}}_{{\text{Na}}^{+}}$ < ${\text{C}}_{{\text{Cl}}^{-}}$ and ${\text{C}}_{{\text{Na}}^{+}}$ > ${\text{C}}_{{\text{K}}^{+}}$ > ${\text{C}}_{{\text{Prt}}^{-}}$, respectively (Figure 2B). This makes the intracellular region electro-positive and the extracellular region electro-negative due to the reversal of concentration gradient (as shown in Figure 2A). The net charge state in the extracellular region (by the virtue of Cl^−^ ions) and in the intracellular region (by the virtue of Na^+^ ions) is represented as, ${\text{Q}}_{{\text{extra}}^{-}}{|}_{{\text{Cl}}^{-}}$ and ${\text{Q}}_{{\text{intra}}^{+}}{|}_{{\text{Na}}^{+}}$ (Figure 2B).

#### 2.3.3. Phase II, Repolarization

During repolarization (Figure 1A), due to the diffusion of K^+^ ions into the extracellular region (Lodish et al., 1995; Purves et al., 2008; Hall, 2010) leaving behind the negatively charged Prt^−^, the ion concentrations in the extracellular and intracellular region change to, ${\text{C}}_{{\text{K}}^{+}}$ > ${\text{C}}_{{\text{Cl}}^{-}}$ > ${\text{C}}_{{\text{Na}}^{+}}$ and ${\text{C}}_{{\text{K}}^{+}}$ < ${\text{C}}_{{\text{Na}}^{+}}$ < ${\text{C}}_{{\text{Prt}}^{-}}$, respectively (Figure 2B). This makes the intracellular region electro-negative and the extracellular region electro-positive due to concentration gradient (as shown in Figure 2A). The net charge state in the extracellular region (by the virtue of K^+^ ions) and in the intracellular region (by the virtue of Prt^−^) is represented as, ${\text{Q}}_{{\text{extra}}^{+}}{|}_{{\text{K}}^{+}}$ and ${\text{Q}}_{{\text{intra}}^{-}}{|}_{{\text{Prt}}^{-}}$, respectively (Figure 2B).

#### 2.3.4. Phase III, Restoration of Pre-depolarization State

Figure 2B represents the refractory period (RF) (Figure 1A) when the Pre-Depolarization state is restored by the Na^+^-K^+^ Pump and the Na^+^, K^+^ ion leak channels (Lodish et al., 1995; Purves et al., 2008; Hall, 2010). Represented by the net charge state as, ${\text{Q}}_{{\text{extra}}^{+}}{|}_{{\text{Na}}^{+}}$ and ${\text{Q}}_{{\text{intra}}^{-}}{|}_{{\text{Prt}}^{-}}$ (Figure 2B). This restores and maintains the electro-chemical gradient ready for next stimulus.

### 2.4. Pressure Schematics

**Figure 3:**
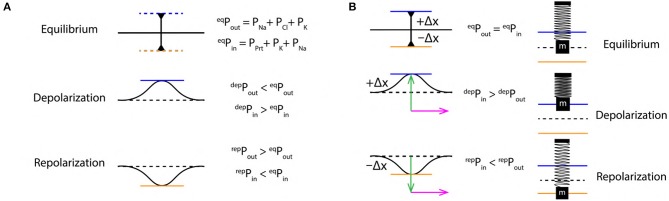
**(A)** Schematics of extracellular (P_out_) and intracellular (P_in_) pressure during, equilibrium (^eq^P), depolarization (^dep^P) and repolarization (^rep^P) states. **(B)** Schematic SM approximation of a small section of nerve membrane during, equilibrium, depolarization and repolarization. Green arrow indicates the force exerted due to variation in PP discussed in section 2.4 and the magenta arrow indicates the direction of propagation of the AP.

The ions are in a state of constant motion/jiggle at room temperature by the virtue of thermal energy (Halliday et al., 1995) in the intracellular and extracellular region. In such state, they can freely interact with any other components (such as proteins, other ions etc.) in the intracellular as well as the extracellular region. This drives the system toward a state of *dynamic equilibrium* due to a balance in the intracellular and extracellular pressure. This state of the system gets perturbed during the event of an AP. At equilibrium (resting state), pressure is balanced on either sides of the membrane i.e., ^eq^P_out_ = ^eq^P_in_ (Figure 3A). In the extracellular region, the pressure is composed of partial pressures (PP) due to Na^+^, Cl^−^ and K^+^ ions (Equation 1). Similarly, the intracellular region is composed of PP due to Prt^−^, K^+^, and Na^+^ ions (Equation 2). The sequence of pressure variation is in concurrence with the ion concentration schematics discussed in previous section 2.3. An AP drives the system away from its dynamic equilibrium state under the influence of the diffusive forces and the ion concentration gradients (Figure 2A) across the membrane (discussed in section 2.3). Then, during depolarization, due to influx of Na^+^ ions in the intracellular region, PP in extracellular region decreases (Equation 3) and increases proportionally in the intracellular region (Equation 4). Where, δ represents a finite change in the PP. Similarly, during repolarization, due to the flow of K^+^ ions into the extracellular region, the PP in intracellular region reduces (Equation 6) and increases PP proportionally in the extracellular region (Equation 5).

(1) Peqout=PNa+PCl+PK

(2) Peqin=PPrt+PK+PNa

(3) Pdepout=Peqout−δPNa

(4) Pdepin=Peqin+δPNa

(5) Prepout=Pdepout+δPK

(6) Prepin=Pdepin+δPK

These changes in PP exert a force perpendicular to the nerve membrane displacing it from its equilibrium position *outward* (during depolarization) and *inward* (during repolarization) (Figure 3A). Such variation in pressure has been discussed previously in experimental studies with plant cells (*Chara braunii* internodes) (Fillafer et al., 2018), this implies that, the justification for variation in pressure provided above is very much plausible.

### 2.5. Fluid Schematics

**Figure 4:**
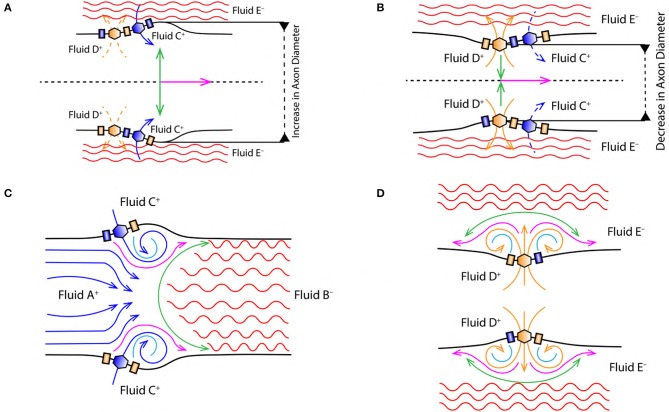
**(A)** Increase in the diameter of axon due to displacement of nerve membrane from its equilibrium position (due to the change in PP, discussed in section 2.4). **(B)** Decrease in the diameter of axon due to displacement of nerve membrane from its equilibrium position (due to the change in PP, discussed in section 2.4). Green arrow indicates the direction of *force*/*stress* exerted perpendicular [outward **(A)** and inward **(B)**] to the nerve membrane and the direction of AP propagation (magenta arrow). Black dashed lines in **(A,B)** represent the cylindrical symmetry, discussed in section 2.2. **(C)** Represents vortex ring formation during the depolarization phase of AP (discussed in section 2.6.2). **(D)** Represents vortex formation during the repolarization phase of AP (discussed in section 2.6.3).

With the thorough background of sections 2.2, 2.3, and 2.4, the increase and decrease in the diameter of nerve membrane accompanying AP are shown in Figures 4A,B due to *coulomb* interactions among the positively charged fluids A^+^, C^+^, D^+^ (section 2.2) and negatively charged fluids B^−^ and E^−^ (section 2.2) during the event of AP discussed in the following section 2.6.

### 2.6. Vortex Formation

Vortex formation will be discussed in the background of theoretical *approximations* and *schematics* disscussed in sections 2.2, 2.3, and 2.4. In the following sections, a *qualitative* analysis of the hypothesis (section 2.1) is established which indicates that, the cascade of events pertaining to formation and propagation of AP create a *vortex ring* of ionic fluid. Vortex ring formation is a physical phenomena generally observed in fluids (liquids/gases) (Shariff and Leonard, 1992). It has been extensively studied from a biological perspective to understand the dynamics of mitral valve and left ventricle of heart (Bellhouse and Bellhouse, 1969; Bellhouse, 1972; Reul et al., 1981; Pasipoularides et al., 1984; Seo et al., 2014). These studies are based on the analysis of fluid dynamics in terms of the velocity vector of the fluid particles. Contrary to this, VT proposed here; is based on the fluid *like* dynamics exhibited by the charged fluids (section 2.2), governed by the coulomb interactions and diffusive forces. This helps to incorporate the ion currents across the membrane which are intricately involved in the initiation and propagation of AP.

#### 2.6.1. Stimuli

The excitatory stimulus (inhibitory stimuli is neglected since it does not lead to an AP) increases the concentration of positive ions Na^+^ in cell body (Lodish et al., 1995; Purves et al., 2008; Hall, 2010). As a result of this, the positive ions start accumulating at the axon hillock as shown in Figure 1C. In accordance with the “*all or none law*” (Adrian, 1914), at the axon hillock graded potentials (Figure 1A) are generated due to the influx of Na^+^ ions. When these graded potentials cross the threshold potential (−55 mV), the Na^+^ ion channels in the close proximity of axon hillock, undergo a conformational change (Na^+^ ion channels open/become active) (Catterall, 1995; Hille, 2001; Catterall et al., 2012). This triggers an AP which manifests itself as a *vortex ring* of charged fluid retaining the *ring* like structure as it travels through the axon (as shown in Figure 4C).

#### 2.6.2. Depolarization

During depolarization (Figure 4C), the positively charged fluid A^+^ (made of Na^+^ ions in the intracellular region) is accelerated toward the axon terminal under the influence of coulomb attraction with negatively charged fluid B^−^ (made of Prt^−^ proteins). Due to the flow of fluid A^+^ into the intracellular region beyond the axon hillock, the voltage gated Na^+^ ion channels get activated (Catterall, 1995; Hille, 2001; Catterall et al., 2012) leading to rapid influx of positively charged fluid C^+^ (made of Na^+^ ions in the extracellular region) driven by diffusive forces governed by the concentration gradient discussed in section 2.3. This event forms along a tri-junction surface of interaction represented by the magenta line in Figure 4C. At a given instance of time in the depolarization phase of AP, these interactions occur between (i) fluid A^+^− C^+^, (ii) fluid A^+^− B^−^ and (iii) fluid C^+^− B^−^. Both, fluid A^+^ and C^+^ are accelerated toward the axon terminal due to the coulomb attraction with fluid B^−^ represented by green surface in Figure 4C. Also, fluid A^+^ and C^+^ interact under coulomb repulsion; pushing fluid C^+^ toward the cell membrane (Figure 4C). Since fluid C^+^ is pushed toward the membrane, it interacts under coulomb repulsion with the diffusing fluid C^+^ along the sky-blue line as shown in Figure 4C. This forms a *ring* like structure (as shown in Figure 4C) which traverses down the axon retaining its shape. The *ring* thus formed is a *vortex* of fluid C^+^ (Na^+^ ions). Hence, the motion of AP is a net effect of repulsion between fluid A^+^− C^+^ and attraction of fluid A^+^− C^+^ with fluid B^−^. This creates a extracellular region dominated by negatively charged fluid E^−^ (made of Cl^−^ ions) and the intracellular region dominated by fluid A^+^ and C^+^ with net positive charge, *depolarizing* (as shown by reversal of ion concentration across the membrane in Figure 2A) the nerve membrane. The influx of fluid C^+^ continues (expanding the axon as shown in Figure 4A) until the membrane reaches a maximum potential (+33 mV) which, causes inactivation of the voltage gated Na^+^ ion channels and activation of the voltage gated K^+^ ion channels (Catterall, 1995; Hille, 2001; Catterall et al., 2012).

#### 2.6.3. Repolarization

Activation of K^+^ ion channels leads to diffusion of fluid D^+^ (made of K^+^ ions in the intracellular region) across the membrane in the extracellular region driven by concentration gradient (as shown in Figure 2B). The extracellular region is dominated by fluid E^−^ due to depolarization (section 2.6.2). Hence, diffusion of fluid D^+^ into the extracellular region is not only driven by the concentration gradient (Figure 2A) but also due to the coulomb attraction with fluid E^−^ along the green line (Figure 4D). The velocity of fluid D^+^ reduces (due to coulomb repulsion with a lower concentrations of fluid C^+^ along the green line as in Figure 4D) as it travels farther from the nerve membrane and the successive diffusion of fluid D^+^ spreads in outward direction along the magenta line governed by coulomb repulsion with fluid D^+^ along the sky-blue line (Figure 4D). The fluid interactions during repolarization phase are hence summarized as interaction between, (i) fluid D^+^− E^−^, (ii) fluid D^+^− D^+^ and (iii) slightly due to fluid D^+^− C^+^. This results in compression of the axon as shown in Figure 4B.

## 3. Membrane Oscillator Theory (MOT)

Thorough *qualitative* analysis performed in section 2 unravels physical changes in the nerve membrane associated with the vortex ring formation in the intracellular and extracellular region. This section deals with, manifestation of physical changes in the nerve membrane as *membrane oscillations*.

### 3.1. Hypothesis

“*Motion of AP is associated with membrane oscillations driven by, vortex ring formation in the intracellular and extracellular region*.”

### 3.2. Assumptions

**Figure 5:**
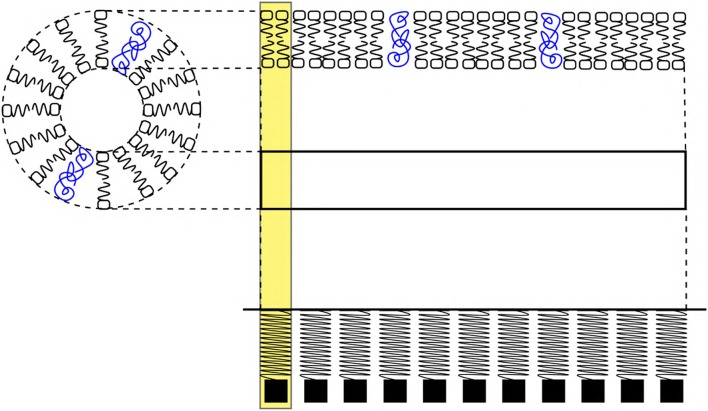
**(Left)** Schematic cross sectional view of an axon. **(Right)** Phospholipid bilayer membrane schematic represents, a section (horizontal view) of the axon which is approximated as an *elastic* ribbon (white strip) which is similar to a linear arrangement of a SM system. The yellow highlight represents a unit length of the membrane approximated as a SM system. The blue colors scribbles represent ion channel proteins. The sections of ribbon are analogous to the SM system.

1. The phospholipid bilayer membrane (Safran, 2018) (nerve membrane) is approximated as an *elastic ribbon* composed of “N” SM systems suspended from a rigid support (Figure 5) (the terms, membrane and ribbon will have synonymous meaning in the discussions hereof).
2. Vortex ring formation (section 2.6) exerts a force perpendicular to the ribbon (as discussed in section 2.4).
3. The displacements in ribbon due to such force is governed by the Hooke's Law (Hooke, 1635–1703).
4. These displacements occur within the *elastic limit* (i.e., stress ∝ strain) (Halliday et al., 1995).
5. These displacements are analogous to the simple harmonic motion of a typical Spring-Mass (SM) system (Figure 3B).
6. Elasticity (k) of the spring indicates *elasticity* of the ribbon which is ideally *constant* for a neuron (although it may vary due to diseases/disorders which are not considered here).
7. The mass (m) of the SM system (discussed in section 3.3) represents the *net* mass of a section of ribbon where the event of AP occurs (Figure 5). This mass is assumed to be constant.
8. A *single cycle* of oscillation performed by the SM system or ribbon corresponds to a *single* event of AP.
9. The spike rate (frequency) of AP (f_AP_) is equal to the frequency of ribbon oscillations (f_ribbon_).
10. Strength of stimuli (E) is directly proportional to the concentration of ions ([C]) in the cell body.

### 3.3. Schematics

**Figure 6:**
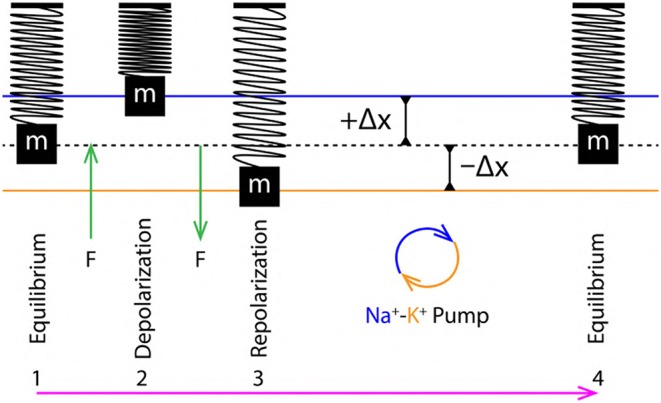
1 to 4 are the sequence of events representing a *single* cycle of membrane oscillation corresponding to a *single* AP.

**Figure 7:**
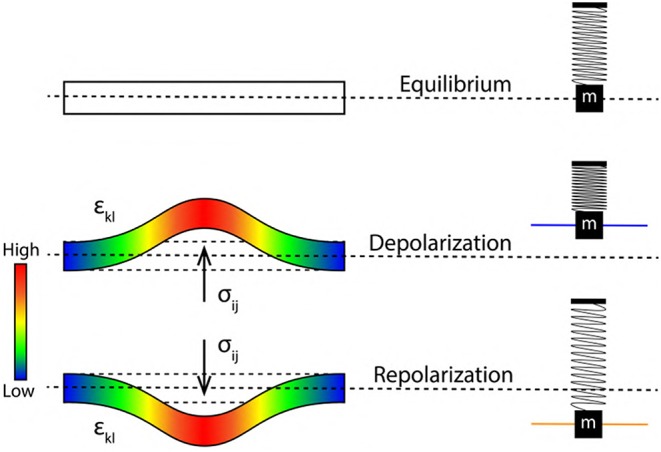
Stress tensor σ_ij_ (bar on left represents the intensity of stress) acting perpendicular to the elastic ribbon during, depolarization and repolarization driving, increment and decrement of axon diameter and pressure. ε_kl_ represents transverse strain tensor leading to increment in axon length. Alongside (right) are corresponding states of SM system.

A SM system typically made up of, a *mass* (m) suspended with the help of a *massless* spring supported by a rigid surface is considered (as shown in Figure 6, 1) (Halliday et al., 1995). At equilibrium, the position of mass (m) is represented by dashed black line (Figure 6, 1). When an external *force* (F) acts on such a mass, it gets displaced by a distance +Δx which is proportional to the applied force (F), Equation (7). Then, according to Hooke's Law (Hooke, 1635–1703), the force and displacement are related as in Equation (8) where, *k* is a constant of proportionality which defines *elasticity* of the spring. The sign in Equation (8) is positive (+) during depolarization (due to displacement +Δx) and negative during repolarization (due to displacement −Δx). These displacements are discussed in terms of the *stress* (perpendicular) and *strain* (transverse) (Figure 7) due to push (/pull) and stretch in the ribbon, respectively (discussed in section 3.4).

(7) F∝ Δx

(8) F= ± k Δx

From section 2.4, the variation of PP in the extracellular and intracellular region creates a *to* and *fro* motion of the nerve membrane (Figure 3A). This motion can be decomposed into three phases (Figure 6) of AP (Figure 1A), (i) Equilibrium, (ii) Depolarization and (iii) Repolarization. According to the variation in PP, at equilibrium ^eq^P_out_ is equal to ^eq^P_in_. This state changes rapidly during depolarization since, ^dep^P_in_ > ^dep^P_out_. The increment of pressure in the intracellular region exerts a force (F) perpendicular to the membrane which displaces it by +Δx about its equilibrium position in outward direction, this is represented by *compression* of the SM system (Figure 6, 2). Similarly, during repolarization, the pressure in extracellular region changes rapidly as ^rep^P_in_ < ^rep^P_out_. The increment of pressure in the extracellular region exerts a force (F) perpendicular to the nerve membrane displacing it by −Δx about its equilibrium position but in opposite direction (inward) as compared to that during depolarization. This is represented by *expansion* of the SM system (Figure 6, 3). Since it is approximated (section 3.2) that, the membrane displacements lie within the elastic limits, it ensures that, the membrane does not rupture due to such rapid changes in pressure which occur on a time scale of milliseconds.

### 3.4. Oscillations

With reference to previous section 3.2, a quantitative analysis describing membrane oscillations is discussed below in terms of changes in the elastic ribbon (Figure 5). Also, the physical origin of spike rate (frequency) of AP is discussed, relating it to the frequency of ribbon oscillations (f_ribbon_). This unravels for the very first time, a mechanism by which we can interpret encoding and transmission of information in a neuron. Figure 6 represents a *single* cycle of membrane oscillation as discussed in section 3.3. Since, these oscillations are governed my the Hooke's Law, the *restoring force* acting on the system is analogous to the activation of Na^+^−K^+^ pump which restores the membrane potential back to the resting state (Figure 1A). Thus, the time taken in transition from phase 3 to 4 represents the refractory period (Figure 6). This period depends on the *elasticity* of membrane defined by, the constant of proportionality in Equation (8) which is assumed to be invariant (section 3.2). Since, a ribbon (nerve membrane) is an example of *continuous* media (Sedov, 1997), the terms in Equation (8) are replaced by second order tensors for continuous media as in Equation (9). Figure 5 represents the *elastic ribbon* approximation of the phospholipid bilayered nerve membrane which is subjected to stress (σ_ij_) analogous to the force (F) in Equation (8), this leads to transverse strain (±ε_kl_) in the ribbon analogous to displacement (±Δ*x*) in Equation (8). Similar to the constant of proportionality in Equation (8), *c*_ijkl_ (i, j = 1, 2, 3) in Equation (9) is a fourth order *stiffness*/*elasticity* tensor. Figure 7 represents a elastic ribbon under application of a perpendicular stress, leading to transverse strain.

(9) $$\begin{array}{l} \sigma_{\text{ij}}=\pm \overset{3}{\underset{\text{k}=1}{\sum }}\overset{3}{\underset{\text{l}=1}{\sum }}{\text{c}}_{\text{ijkl}}\varepsilon_{\text{kl}} \end{array}$$

**Figure 8:**
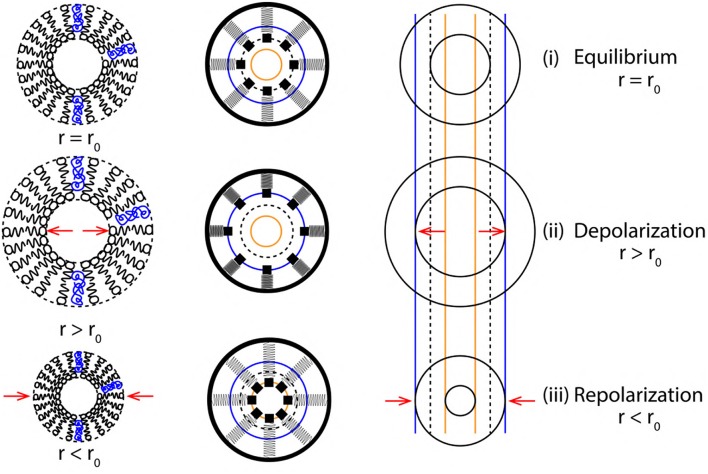
Cross sectional view (top to bottom) of membrane oscillations during a single cycle of AP. (left to right) Membrane approximations (phospholipid structure > SM system > elastic ribbon), representing membrane oscillations in terms of variation of axon radius (r) about its equilibrium position (r_0_).

Figure 8 represents a cross sectional view of the membrane oscillations with variation in axon radius (r = r_0_ at equilibrium) as r > r_0_ during depolarization and r < r_0_ during repolarization. A *single* event of AP (depolarization, +Δx and repolarization, −Δx) are represented by the variation of axon diameter approximated as a ribbon with the corresponding SM states. Since the two, elastic ribbon and SM system are analogous, the frequency of ribbon oscillation f_ribbon_ represents the spike rate of AP (section 3.2). Based on the approximations of section 3.2, the elasticity (k) and mass (m) of a unit length of ribbon (Figure 5) are constant, this makes the frequency of oscillations dependent indirectly on k and m. Due to this constrain we cannot establish a relation between the spike rate (frequency) of AP and strength of stimuli directly. This is solved in the following section, in order to establish a correlation between the strength of stimuli and spike rate of AP.

### 3.5. Frequency

**Figure 9:**
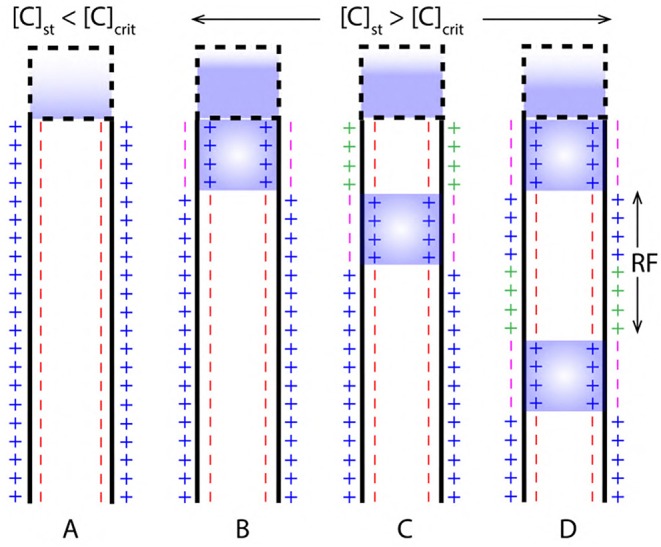
**(A)** Concentration of ions due to stimuli [C]_st_ in the cell body is less than the critical concentration [C]_crit_ required to generated an AP. **(B,C)** AP initiation and propagation when, the [C]_st_ is greater than [C]_crit_. **(D)** Two successive events of AP resolved by a refractory period (RF).

The strength of stimuli (E) and the corresponding concentration of ions ([C]) in the cell body are related as in Equation (10) based on the assumptions of section 3.2. This implies that, if a neuron is stimulated for time (*t*) then, the concentration of ions in the cell body increases due to the activation of ion channels (which can be, voltage, ligand and mechanically gated). A *critical concentration* ([C]_crit_) of ions in cell body corresponds to, the value of threshold potential (Figure 1A) and is necessary for the initiation of an AP (Figure 9). Then, the critical strength of the stimuli E_crit_ defines, a threshold strength of stimuli required for a successful initiation of AP.

(10) $$\begin{array}{l} \text{E}\propto \left[\text{C}\right] \end{array}$$

During depolarization, the concentration of ions in cell body due to stimuli [C]_st_ exceeds [C]_crit_ (as in Figure 9B). Then, a relation between *net* concentration of ions due to stimuli ^net^[C]_st_ and [C]_crit_ can be established as in Equation (11) where, β is a multiplication factor.

(11) [C]netst= β [C]crit

**Figure 10:**
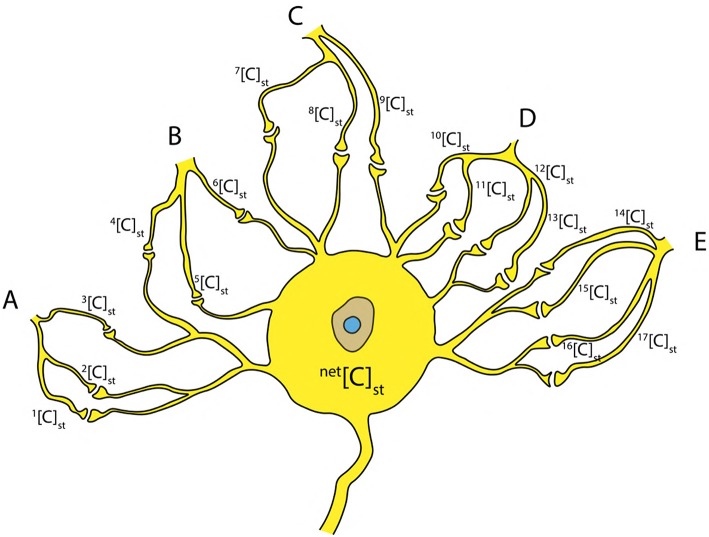
Inputs from neighboring neurons **(A–E)** to the cell body resulting in a net concentration due to stimuli ^net^[C]_st_ at the axon hillock.

Since it is known that, an AP is generated by recurrent attempts of graded potentials (Figure 1A) (Lodish et al., 1995; Hall, 2010), the *net* concentration ^net^[C]_st_ due to the stimuli from neighboring neurons (A, B, C, D, E) (Figure 10) can be written as a sum of individual concentrations as in Equation (12) (Figure 10). This can be generalized for *k* inputs (as in Equation 13). Hence, the concentration ^net^[C]_st_ can be written as a linear combination (using Equation 11) of critical concentration [C]_crit_ (as in Equation 14) such that, it has *one to one* correspondence with the terms in Equation (13).

(12) [C]netst=[C]1st +[C]2st + ... +[C]17st

(13) [C]netst=[C]1st +[C]2st + ... +[C]kst

(14) [C]netst=β1 [C]crit+β2[C]crit+...+βk[C]crit=∑i=1kβi[C]crit

Multiplication factor β (in Equation 11) is decided by the *ratio* of, *net* concentration ^net^[C]_st_ and the critical concentration [C]_crit_ as in Equation 15. This factor decides the *number* of AP (or spike rate of AP) for a given stimuli which is nothing but, the frequency f_ribbon_ (as in Equation 16). It can be deduced that, the concentration ^net^[C]_st_ is a factor which decides the frequency of oscillations f_ribbon_ (i.e., spike rate of AP). This is justified by an illustration, consider a stimuli which acts on a neuron for time (t) such that, the concentration ^net^[C]_st_ is **100** mmol/L, then, for a critical concentration (required to generate an AP) say [C]_crit_ = **20** mmol/L, the multiplication factor β turns out to be **5**. Therefore, according to Equation (16), the frequency f_ribbon_ should be ≃ 5 per unit time (*t*). It turns out that, the frequency f_ribbon_ is governed by RF (which depends on the functions of Na^+^-K^+^ pump, Na^+^, K^+^ ion leak channels, Figure 1A) and can vary with respect to constant of elasticity (k) of the ribbon [i.e., elasticity of the nerve membrane discussed in Heimburg (2009)].

(15) β =[C]netst[C]crit

(16) $$\begin{array}{l} \beta \propto {\text{f}}_{\text{ribbon}} \end{array}$$

The *periodic time* (Halliday et al., 1995) of oscillations (T) is a function of RF i.e., T_RF_. It is related to the constant of elasticity (k), net mass (m) of a section and the frequency of ribbon oscillations f_ribbon_ as in Equations (17) and (18), respectively.

(17) $$\begin{array}{l} {\text{T}}_{\text{RF}}=2\pi \sqrt{\frac{\text{m}}{\text{k}}} \end{array}$$

(18) $$\begin{array}{l} {\text{f}}_{\text{ribbon}}=\frac{1}{{\text{T}}_{\text{RF}}} \end{array}$$

Then, the frequency f_ribbon_ is related to the net strength of the stimuli ^net^E_st_ as in Equation (19) (obtained using Equations 10, 15, and 16).

(19) fspike ~ fribbon = 12πkm ∝[C]netst[C]crit

Hence, for a unit length of the nerve membrane which is approximated as an elastic ribbon and SM system (Figure 5), the frequency f_ribbon_ has a dependence on the strength of stimuli (in terms of ion concentrations [C]) as in Equation (19). This frequency is the spike rate of AP represented by f_spike_.

## 4. Discussion

The major question persistent in the field of neurobiology is, ***how***do neurons *encode* information and transmit it across the axon as an AP? How is the strength of stimuli correlated to the spike rate of AP? What is the physiological role of the nerve membrane in this regard?

Although, the HH model (Hodgkin and Katz, 1949; Hodgkin and Huxley, 1952a,b; Hodgkin, 1964a,b) treats nerve membrane in a purely electric regime, it does not justify the key physiological features which is the reason for its criticism (Drukarch et al., 2018). But, the *correlation* between strength of stimuli and the spike rate of AP (which is how cells encode information) in terms of *variation* in the input/stimuli current can be easily established from the HH model. Prior to the pioneering work by HH, Wilke (1912) had proposed a Pressure Wave theory for AP, indicating toward the physiological changes in nerve membrane successive to an AP event but, missed out on correlating the strength of stimuli and the spike rate of AP. Also, the theory proposed by Wilke did not gain much of attention until, the advent of sophisticated experimental techniques which proved that, the AP is accompanied with certain physiological changes (Hill, 1950; Abbott et al., 1958; Julian and Goldman, 1962; Biondi et al., 1972; Hill et al., 1977; Iwasa and Tasaki, 1980; Iwasa et al., 1980; Tasaki and Iwasa, 1982; Terakawa, 1985; Tasaki et al., 1989; Tasaki and Byrne, 1992a,b; Galbraith et al., 1993; Tasaki, 1999). These interpretations are polarized into two school of thoughts, one which revolved around the electrical interpretation and the other around non-electrical interpretation. This bifurcation continued, until recently when, new theories were proposed to address the empirical observations by combining the electrical features with non-electrical (mechanical/thermodynamic) features of the nerve membrane pertaining to AP. ST by Heimburg-Jackson (Heimburg and Jackson, 2005, 2007), Rvachev model (Rvachev, 2010), and MSW interpretation by Hady-Machta (El Hady and Machta, 2015) have resulted in successful incorporation of the physiological changes accompanying AP. These models present a mixed interpretation of nerve membrane i.e., ST model (Heimburg and Jackson, 2005, 2007) addresses mechanical and thermodynamic nature of nerve membrane whereas, Rvachev and MSW model indicate toward the interplay of electrical and mechanical nature of nerve membrane. This “*hit and miss*” scenario necessitates the development of a model which can unify them and establish a *correlation* between the strength of stimuli and spike rate (frequency) of AP (which is purely electrical and fundamental to every neural activity).

A novel approach is proposed in this paper to unify the well established theories encompassing delicate and important features of nerve membrane from a thorough biophysical and physiological perspective. From the VT it is suggested that, the *vortex ring* formation leads to variation in intracellular and extracellular pressure indicating that, the motion of AP through the axon of a neuron is *peristaltic* in nature which, justifies the increase and decrease in axon diameter, pressure and length observed empirically. The experimental verification of vortex ring formation is possible by performing high speed patch-clamp fluorometry (generally used to study ion channel conformations Bullen et al., 1997; Harms et al., 2003; Kusch and Zifarelli, 2014; Talwar and Lynch, 2015; Wulf and Pless, 2018) with spatio-temporal accuracy using fluorescent indicators for ion concentrations (Tsien, 1989). As far as the absorption-emission heat and variation in optical properties is concerned, the interpretation from ST, Rvachev and MSW model is retained. The MOT describes, membrane oscillations driven by the vortex ring formation which is used establish *quantitatively*, a *correlation* between the strength of stimuli and the spike rate of AP.

Hence, from a macroscopic (neglecting the complexities of molecular cell biology to some extent) perspective, VT and MOT unifies ST, Rvachev and MSW models. It is concluded that, MOT can be used to develop of novel computational tools to mimic neural activity with better accuracy.

## Author Contributions

The corresponding author has individually developed the theory, illustrations and wrote the manuscript.

### Conflict of Interest

The author declares that the research was conducted in the absence of any commercial or financial relationships that could be construed as a potential conflict of interest.
